# Peritoneal Metastasis of Cholangiocarcinoma Treated with Cytoreductive Surgery and Hyperthermic Intraperitoneal Chemotherapy at the Instituto Nacional de Cancerología, Colombia

**DOI:** 10.7759/cureus.6697

**Published:** 2020-01-18

**Authors:** Dary L Hernandez, Juliana Restrepo, Mauricio Garcia Mora

**Affiliations:** 1 Surgical Oncology, Instituto Nacional de Cancerologia, Bogotá D.C., COL; 2 Surgical Oncology, Instituto Nacional de Cancerología, Bogotá D.C., COL; 3 Breast and Soft Tissue Surgery, Instituto Nacional de Cancerologia, Bogotá D.C., COL

**Keywords:** cholangiocarcinoma, klatskin, peritoneum, hyperthermia, chemotherapy, cytoreduction surgical procedures

## Abstract

Cholangiocarcinoma is a low-frequency neoplasm of onset with a poor prognosis. Peritoneal carcinomatosis is the most frequent site of metastasis with a standard palliative chemotherapy treatment.

In the present article, we describe the case of a 35-year-old woman with peritoneal carcinomatosis secondary to an intrahepatic cholangiocarcinoma who was treated with cytoreductive surgery (CRS) and hyperthermic intraperitoneal chemotherapy (HIPEC) as a non-standard therapeutic method. The patient has disease-free survival of 12 months with very good quality of life.

The treatment of peritoneal metastasis from cholangiocarcinoma by CRS and HIPEC is feasible and could proportion better survival to these patients compared to systemic palliative chemotherapy. These therapeutic modalities can complement each other.

## Introduction

Peritoneal carcinomatosis is defined as the implantation of neoplastic cells in the peritoneum from an intra- or extra-abdominal tumor. Historically, it has been considered a systemic and incurable disease, with a poor short-term prognosis regardless of its origin, which is why it has usually been treated palliatively. Its presence indicates an advanced stage (state IV) of many tumors that develop in abdominal and pelvic organs, with ovarian, stomach and colorectal cancer being the most frequent causes [1-3].

Cholangiocarcinoma represents 3% of malignant tumors originating in the gastrointestinal tract and 15% of those of hepatobiliary origin and its only potentially curative treatment is complete surgical resection with a five-year overall survival (OS), after curative surgery, 30 to 45% [4,5]. The peritoneum is the most frequent site of metastatic disease in a patient with cholangiocarcinoma and is present in 10 to 20% at the time of diagnosis [6]. The standard treatment for unresectable cholangiocarcinoma or with peritoneal dissemination is palliative chemotherapy with gemcitabine and cisplatin, and the median OS for these patients is 15.2 months [7,8].

The management of the disease is a challenge for its few therapeutic options in advanced stages and for poor survival, hence the search for more radical regional approaches through cytoreduction (CRS) and hyperthermic intraperitoneal chemotherapy (HIPEC) as is the case we present.

## Case presentation

**Figure 1:**
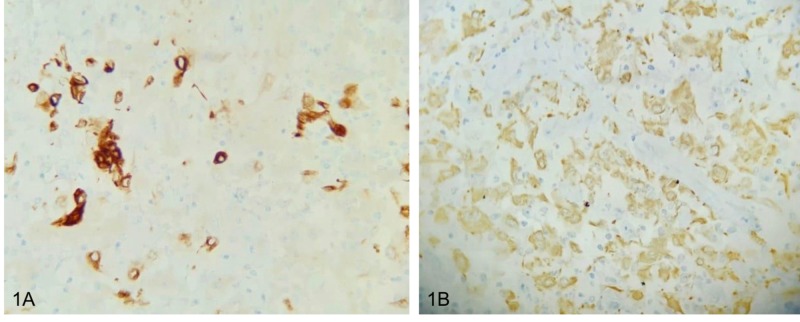
(A) Immunohistochemistry cocktail cytokeratins AE1 and AE3, 40X. (B) Immunohistochemistry CK7, 40X.

**Figure 2:**
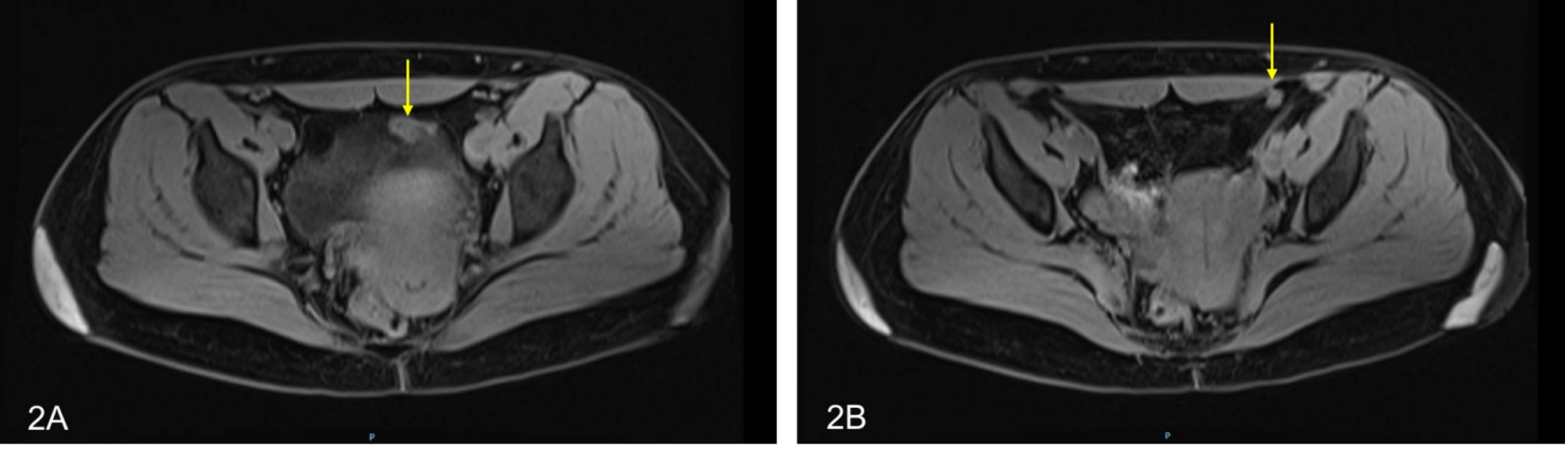
(A) Solid aspect peritoneal implant in close contact with the left anterior margin of the bladder measuring 14 x 26 mm, intermediate signal in T2. (B) Solid aspect peritoneal implant in close contact with the lateral and posterior margin of the left major rectus of the abdomen measuring 7.2 x 15.3 mm, intermediate signal in T2.

We present a 35-year-old patient referred to the National Cancer Institute (INC) with a diagnosis of intrahepatic cholangiocarcinoma who was taken to another institution for central hepatectomy plus omentum biopsy and mass resection in the right iliac fossa by peritoneal implants in these two locations. Extrainstitutional pathology studies reported a malignant epithelial tumor which was consistent with adenocarcinoma but suggested additional immunohistochemical studies to define the origin of the primary tumor. Gallbladder studies reported chronic cholecystitis and peritoneal tissue and omentum were positive for malignancy. The subsequent immunohistochemical study reported a moderately differentiated adenocarcinoma whose morphology and immuno-profile favors a cholangiocarcinoma positive for AE1 (Figure 1A), CK7 (Figure 1B), CK19 and focally for CA 19 9; and negativity for chromogranin, synaptophysin, carcinoembryonic antigen, CDK20, CDX2 and HEP-PAR. A positron emission tomography reported a soft tissue multilobulated lesion, located in the superior and anterior aspect of the bladder, some of them with metabolic increase with standardized uptake value (SUV) of 5. The service of oncology treatment initiated palliative chemotherapy with gemcitabine plus cisplatin and decided to refer to the peritoneal malignancy service of the National Cancer Institute to define the possibility of treatment with CRS and HIPEC. Re-staging was requested with a contrasted resonance of the pelvis that reported lesions compatible with peritoneal implants in the minor pelvis (Figure 2A, 2B ) and two cystic mucinous lesions in the tail of the pancreas; abdominal and thoracic tomography images did not report distance involvement. Case was discussed at the board of peritoneal tumors and it was decided to take cytoreduction surgery plus HIPEC as the best therapeutic option. During the surgical procedure, a peritoneal carcinomatosis index (PCI) of 4 was found for implants in the 1.5-cm bladder dome, 5-mm implants in both ovaries and uterus, and 5- and 8-mm peritoneal implants in greater omentum. The surgical treatment was parietal peritonectomy, distal pancreatectomy, total omentectomy, radical appendectomy, hysterectomy, bilateral salpingo-oophorectomy and intraperitoneal hyperthermic chemotherapy with cisplatin 90 mg/m^2^ + doxorubicin 15 mg/m^2^ at 41°C. The pathology report of the surgical specimen describes compromise due to large cell carcinoma with large eosinophilic cytoplasm, which probably causes metastatic carcinoma. During 12 months of follow-up with abdominal resonances every three months and chest tomography every six months, no evidence of local or distant relapse has been documented.

## Discussion

Peritoneal carcinomatosis is the presence of tumor dissemination in the peritoneal cavity, from an intra- or extra-peritoneal neoplasm, being a complex biological process that involves cell proliferation, rupture or perforation of the serosa or extension from the rupture of regional tumor nodes, evasion of the immune system, adhesion to the site of metastases, mesothelial translocation and growth at the site of metastases [9,10]. The tumors that most frequently cause this condition are colon cancer, gastric cancer, ovarian cancer and peritoneal pseudomyxoma.

Systemic chemotherapy is usually the treatment for unresectable or metastatic cholangiocarcinoma with a combination of gemcitabine and cisplatin, among other possible combinations. Valle et al. established this regimen as the gold standard in 2010, where they described a median OS of 11.7 months significantly longer than the 8.1 months obtained with gemcitabine alone [11]. However, in some selected patients the treatment of peritoneal carcinomatosis secondary to cholangiocarcinoma with CRS and HIPEC has been described. In 2018, Amblard et al. conducted a retrospective study that included 34 patients with biliary cancer who were taken to CRS and HIPEC and compared them with 21 patients who were treated with chemotherapy alone, the OS was 21.4 and 9.3 months respectively and the three-year OS was 30% for the group treated with CRS and HIPEC and 10% for the group of patients treated with chemotherapy. According to the variables analyzed, the candidates who benefit most from CRS and HIPEC are young patients, with good functional status, peritoneal carcinomatosis due to gallbladder carcinoma, low PCI and susceptible to complete cytoreduction. More than 50% of patients in both groups had a progression at 12 months and the median progression-free survival was not different in both groups, 9.3 months for the chemotherapy group versus 9.3 months for the CRS group and HIPEC with a p-value of 0.479 [12].

In Colombia, the first cases of cytoreductive surgery and HIPEC were reported for the treatment of patients diagnosed with peritoneal pseudomyxoma in 2009 [13]. In 2012, a series of 24 patients with various pathologies were reported, and in 2017 two cases of second cytoreduction were reported in patients with peritoneal pseudomyxoma [14,15].

At the National Cancer Institute of Colombia there is a program of peritoneal malignancy since 2013 and more than 150 patients with peritoneal pathology secondary to colon, appendix, ovarian, gastric and peritoneal pseudomyxoma have been treated, but additionally, unconventional pathologies such as peritoneal mesothelioma have also been treated, and this case we previously presented with a cholangiocarcinoma with a good result related to a disease-free survival superior to that reported in the literature [16].

## Conclusions

The usual treatment for unresectable or metastatic cholangiocarcinoma is palliative chemotherapy. To our knowledge, this is the second case report of metastatic to peritoneal cholangiocarcinoma treated with CRS and HIPEC. Although it is a non-standard management, in this case a favorable oncological result was achieved with acceptable morbidity. Currently the patient is free of disease after 12 months of follow-up, exceeding the known median that offers systemic chemotherapy, which may be associated with factors such as age, low carcinomatosis index and complete cytoreduction. More prospective studies are required that include a greater number of patients to validate these results.
